# SWOffinder: Efficient and versatile search of CRISPR off-targets with bulges by Smith-Waterman alignment

**DOI:** 10.1016/j.isci.2023.108557

**Published:** 2023-11-23

**Authors:** Ofir Yaish, Amichai Malle, Eliav Cohen, Yaron Orenstein

**Affiliations:** 1 School of Electrical and Computer Engineering, Ben-Gurion University the Negev, Beer Sheba 8410501, Israel; 2 Department of Computer Science, Bar-Ilan University, Ramat Gan 5290002, Israel; 3 The Mina and Everard Goodman Faculty of Life Sciences, Bar-Ilan University, Ramat Gan 5290002, Israel

**Keywords:** Techniques in genetics, Bioinformatics, Biological constraints, Algorithms

## Abstract

CRISPR/Cas9 technology is revolutionizing the field of gene editing. While this technology enables the targeting of any gene, it may also target unplanned loci, termed off-target sites (OTS), which are a few mismatches, insertions, and deletions from the target. While existing methods for finding OTS up to a given mismatch threshold are efficient, other methods considering insertions and deletions are limited by long runtimes, incomplete OTS lists, and partial support of versatile thresholds. Here, we developed SWOffinder, an efficient method based on Smith-Waterman alignment to find all OTS up to some edit distance. We implemented an original trace-back approach to find OTS under versatile criteria, such as separate limits on the number of insertions, deletions, and mismatches. Compared to state-of-the-art methods, only SWOffinder finds all OTS in the genome in just a few minutes. SWOffinder enables accurate and efficient genomic search of OTS, which will lead to safer gene editing.

## Highlights

• SWOffinder finds off-target sites efficiently and accurately by SW-local alignment
• SWOffinder enables versatile search thresholds of the number of mismatches and bulges
• SWOffinder outperforms other off-target site search methods in runtime and accuracy
• SWOffinder will lead to enhancements in the safety of gene-editing applications

## Graphical abstract

**Figure:**
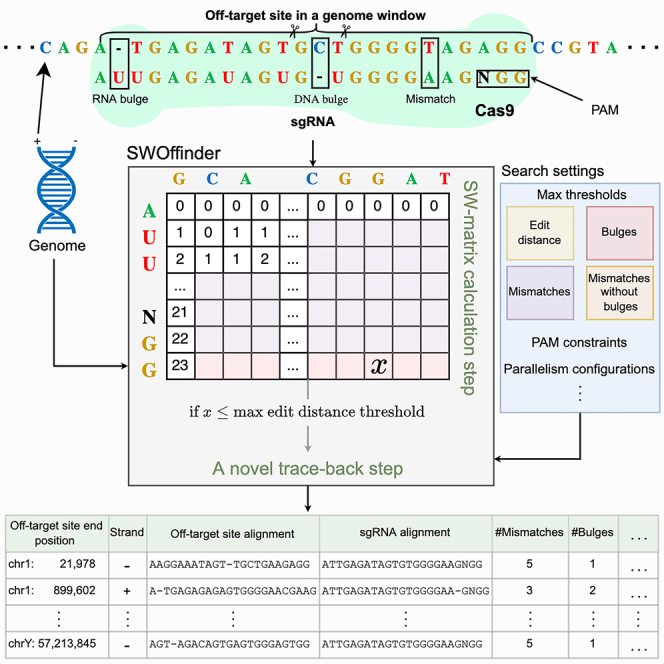

## Introduction

CRISPR/Cas9 has revolutionized gene-editing research and applications.^1^ CRISPR/Cas9 enables the precise and efficient modification of DNA sequences in living cells by a single-guide RNA (sgRNA) targeting specific loci of 20nt followed by a protospacer adjacent motif (PAM) sequence, which is NGG for Cas9. CRISPR/Cas9 can be used in a wide range of organisms, from bacteria to humans, and it can be applied in a variety of different fields, including agriculture and medicine.

**Figure 1:**
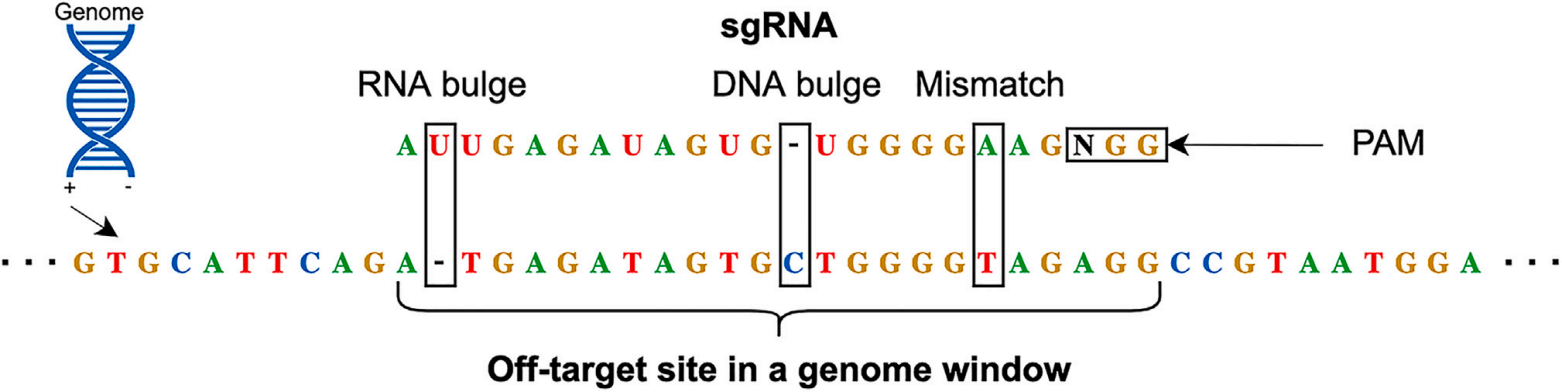
Off-target site alignment illustration An off-target site alignment with 1 DNA bulge, 1 RNA bulge, and 1 mismatch of a sgRNA sequence AUUGAGAUAGUGUGGGGAAG with NGG PAM and a genome window of the forward strand.

Unfortunately, there are concerns about the safety of using CRISPR/Cas9.^2^ One of the main risks associated with CRISPR/Cas9 is the possibility of off-target sites. Off-target sites occur when the CRISPR/Cas9 system edits unintended regions in the genome, which can lead to undesired consequences, such as the introduction of new mutations or the disruption of important genes. Off-target sites often occur in regions that are similar by sequence, i.e., in a small Hamming or edit distance, to the sgRNA (Figure 1).

Off-target search considering only mismatches, i.e., up to some Hamming distance, has been solved efficiently by a plethora of computational methods, with the most popular being Cas-OFFinder.^3^ But, the efficient search for potential off-target sites up to some edit distance, i.e., when considering mismatches, insertions, and deletions, is still an open problem. It has been shown that the problem of computing the edit distance between two strings cannot be computed in subquadratic time.^4^ Nonetheless, several methods have been developed for CRISPR off-target search considering insertions and deletions, termed DNA and RNA bulges, including CRISPRitz,^5^ CALITAS,^6^ and an unpublished variant of Cas-OFFinder.

Unfortunately, these methods fail to find all potential off-target sites in a feasible runtime, report incomplete lists of off-target sites, and/or do not support versatile threshold criteria. While CALITAS overcomes the most prominent limitations of Cas-OFFinder and CRISPRitz (the exponential dependence of runtime in the number of gaps, allowing only DNA or RNA insertions, and reporting all possible alignments^6^), its runtime is prohibitive, especially when running it on thousands of sgRNAs in parallel, which is required in most sgRNA-design scenarios.^7^ Moreover, CALITAS searches for off-target sites with specific scores for mismatches, insertions, and deletions, while common applications require searching for off-target sites with a separate threshold for each edit operation.^8^ As a result, there is currently no feasible solution to the CRISPR off-target search problem, when considering insertions and deletions and a separate threshold for each edit operation.

**Figure 2:**
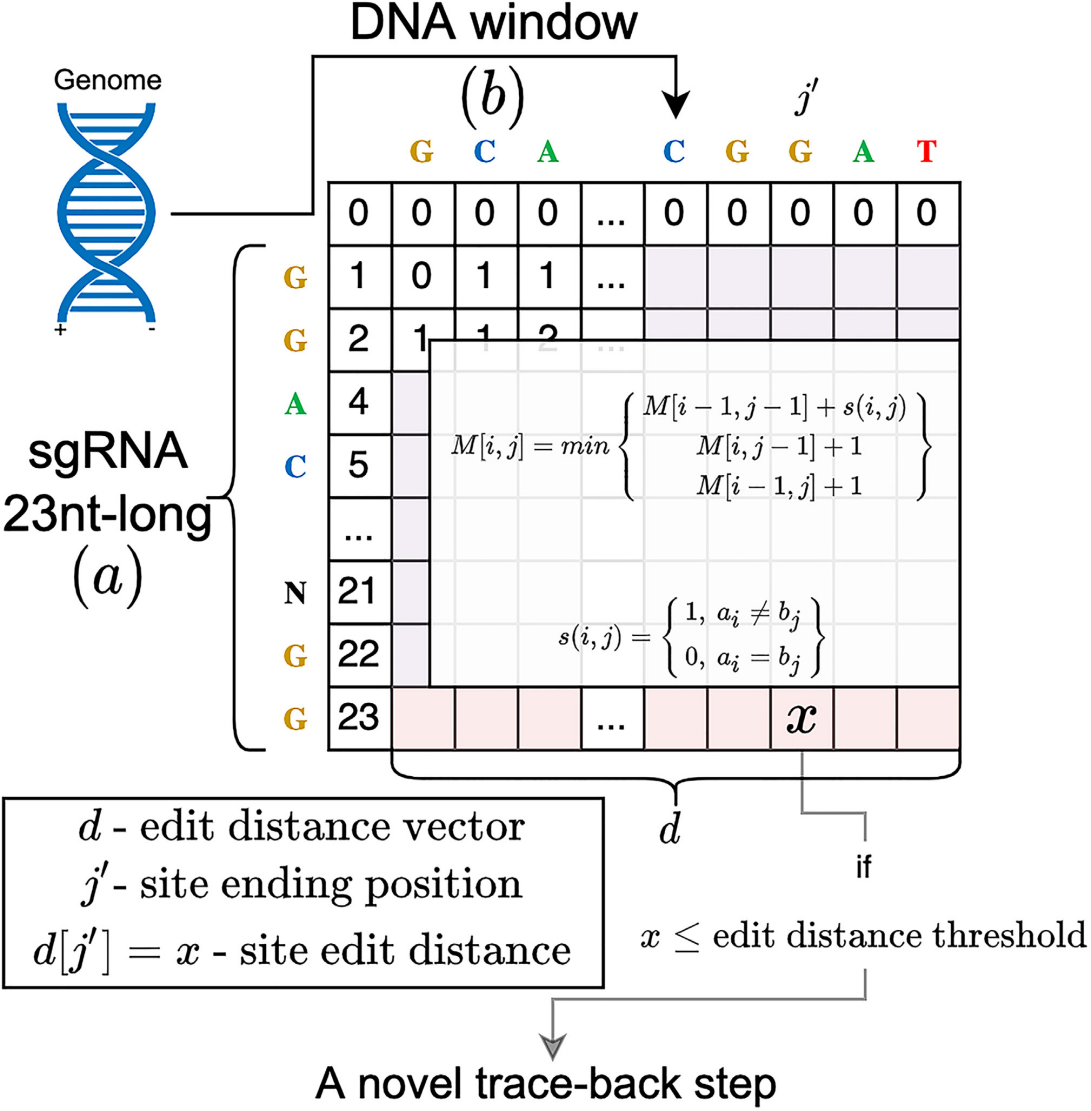
Overview of SWOffinder for searching off-target sites across a given genome First, in the SW-matrix calculation step, SWOffinder scans the genome for all end positions of sites with an edit distance that meets a user-specified threshold using a novel version of the Smith-Waterman alignment algorithm. Then, in the trace-back step, SWOffinder applies a novel recursive procedure on the subset of end positions obtained by the SW-matrix calculation step to filter sites, which do not meet the user-specified operation-specific thresholds, and find their alignment that meets those thresholds with minimum edit distance (Algorithm 1).

Here, we developed SWOffinder, a new highly efficient method to find all off-target sites up to some edit distance, which is based on the classic Smith-Waterman (SW) alignment (Figure 2). We implemented a novel trace-back method to find off-target sites under versatile criteria, such as separate limits on the number of bulges and mismatches. Our results compared to state-of-the-art methods show that SWOffinder finds all off-target sites in the human genome in only a couple of minutes. We expect our method to enable accurate and efficient genomic search of potential off-target sites, which will make gene-editing applications safer.

Algorithm 1 **The trace-back step of SWOffinder to post-process off-target sites**

**Input** DP matrix (*M*), sgRNA (*a*), Genome window (*b*), sgRNA pointer index (*i*), Genome-window

pointer index (*j*), Mismatch count (mc), Bulge count (bc), Partial alignment - sgRNA (pa), Partial

alignment - DNA (pb)

**Output** Alignment that follows the user-specified thresholds with minimum edit distance

**Initialization**i= sgRNA length, j= matching end position, mc=bc=0, pa=pb=ϵ *ϵ* (empty string),

 edits, mismatches, and bulges thresholds (maxE,maxM,maxB,maxMB) are pre-defined

1: **procedure** Post(M,a,b,i,j,mc,bc,pa,pb)

2:  **if** mc or bc are greater than the mismatches or bulges thresholds, respectively **then**

3:  **return** null         ▷ Could not find alignment - returns null

4:  **end if**

5:  **if** i=0 **then**

6:  **return** (pa,pb,mc,bc)       ▷ Found alignment - returns alignment object

7:  **end if**

8:  **if** j=0 **then**

9:  **return** null          ▷ Could not find alignment - returns null

10:  **end if**

11:  effMaxE←maxEifbc=0elsemin(maxE,maxB+maxMB)    ▷ Calculate effective max edit distance

12:  **if** M[i][j]>(effMaxE−mc−bc) **then**

13:  **return** null          ▷ Could not find alignment - returns null

14:  **end if**

15:  mp←0ifa[i−1]=b[j−1]else1        ▷ Mismatch penalty

16:  mAlignment←Post(M,a,b,i−1,j−1,mc+mp,bc,pa+a[i−1],pb+b[j−1])    ▷ Alignment without bulge at position

17:  $aBulgeAlignment\leftarrow \text{Post}\left(M,a,b,i-1,j,mc,bc+1,pa+a\left[i-1\right],pb{+}^{\prime \prime }{-}^{\prime \prime }\right)$     ▷ Alignment with sgRNA bulge

18:  $bBulgeAlignment\leftarrow \text{Post}\left(M,a,b,i,j-1,mc,bc+1,pa{+}^{\prime \prime }{-}^{\prime \prime },pb+b\left[j-1\right]\right)$     ▷ Alignment with DNA bulge

19:  **if** mAlignment=aBulgeAlignment=bBulgeAlignment=null **then**

20:  **return** null ▷          ▷Could not find alignment - returns null

21:  **end if**

22:  **return** The alignment with minimum bulges and mismatches among

  mAlignment, aBulgeAlignment, and bBulgeAlignment

23: **end procedure**

## Results

To evaluate both off-target site identification and runtime performance of SWOffider and existing methods, we used as a test case the set of chromosomes derived from the hg38 reference genome and the sgRNA AUUGAGAUAGUGUGGGGAAG with NGG PAM which was also used in CALITAS becnmarks.^6^ We present results with the NGG PAM, which fits CRISPR/Cas9, the most popular nuclease, and note that SWOffider can run on sgRNAs of any length and any PAM. One of the key strengths of SWOffider is its versatile search options, which are particularly suited for common use cases. However, since existing methods do not support such versatile search options, we compared them to SWOffider only over their search options.

### Comparison of the number of identified sites and runtime at an edit-distance threshold

We compared SWOffidner to CALITAS and CRISPRitz in the number of identified off-target sites with up to 4 edits and in a single-threaded runtime. The specific parameters of this run for the different methods were.

(1) SWOffinder: maxE=maxM=maxMB=maxB=4 without PAM edits allowed.
(2) CALITAS: Up to 4 edits without PAM edits allowed.
(3) CRISPRitz: Up to 4 mismatches and 4 bulges with an index file for NGG PAM. Note that CRISPRitz has the inability to search up to 4 edits directly, so we filtered the obtained sites to the desired edit threshold.

**Figure 3:**
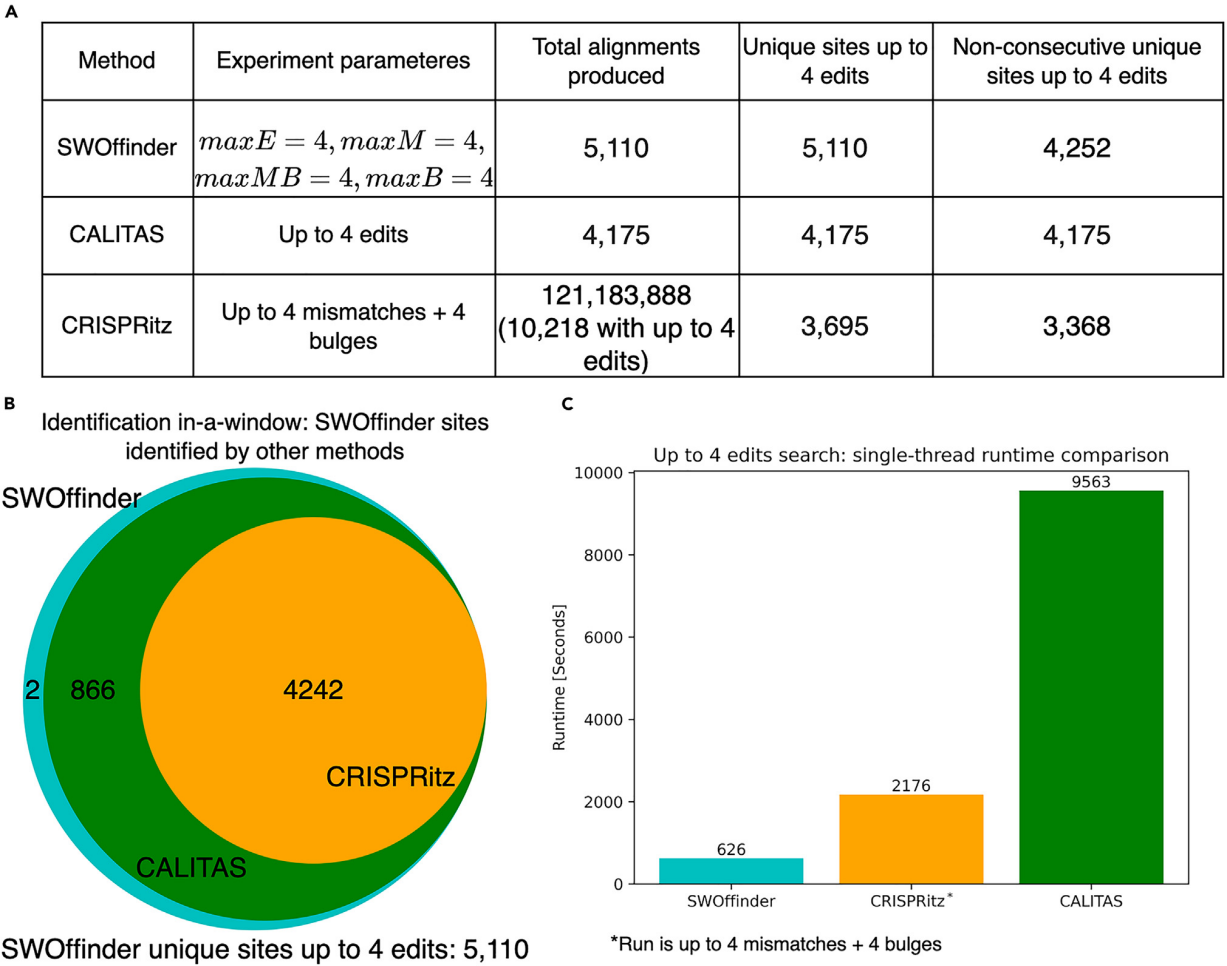
Off-target sites identification and runtime comparison of SWOffinder, CALITAS, and CRISPRitz (A) Comparison of the number of sites found by each method. We ran SWOffinder and CALITAS to find off-target sites up to edit distance 4, and CRISPRitz with up to 4 mismatches and 4 bugles (as it does not support an edit-distance threshold). (B) The overlap between sites that were identified by SWOffinder and sites that were found by CALITAS and CRISPRitz in a window. SWOffinder found all sites that were found by CALITAS and CRISPRitz. (C) Runtime comparison of SWOffinder, CALITAS, and CRISPRitz in searching off-target sites up to edit distance 4. CRISPRitz was run with up to 4 mismatches and 4 bulges (as it does not support an edit-distance threshold).

Regarding off-target site identification, SWOffinder found the greatest number of unique off-target sites (Figure 3A). While the total number of alignments produced by CRISPRitz is the greatest (10,218 when filtering the results to up to 4 edits), this number is the greatest due to the redundancy in the number of alignments for the same off-target site. After removing redundant sites, we discovered that CRISPRitz found only 3,695 unique sites compared to 4,175 unique sites found by CALITAS and 5,110 by SWOffinder.

While SWOffinder enables the user to choose whether to output all possible off-target sites or optimal sites within a user-defined genome window, CALITAS is limited to the second option due to its use of semi-global alignment, where the default window size is 1000, which may lead to missing proximal off-target sites. Therefore, we compared the number of non-consecutive unique sites and performed an identification in-a-window test (for definitions, see Subsection Benchmarking off-target sites identification). SWOffinder found the greatest number of non-consecutive unique off-target sites with 4,252 sites compared to 4,175 and 3,368 by CALITAS and CRISPRitz, respectively (Figure 3A). In the identification in-a-window test, only 4,242 out of the 5,110 unique sites found by SWOffinder could be associated with CRISPRitz (Figure 3B). When comparing CALITAS and SWOffinder in this test, we found that 2 of the sites found by SWOffinder could not be associated with CALITAS. The results show the supremacy of SWOffidner in identifying all potential off-target sites in various settings.

Regarding the runtime in a single-threaded run, SWOffinder’s runtime is 626 seconds, which is significantly faster than CALITAS with a runtime of 9,563 seconds (Figure 3C). In this experiment, SWOffinder was also faster than CRISPRitz, which ran in 2,176 seconds. We note that the last is not a direct comparison since CRISPRitz cannot search up to 4 edits directly. In this experiment, the maximum memory usage of SWOffinder and CALITAS were very similar, at 2.3GB and 2.1GB, respectively. CRISPRitz had maximum memory usage of 5GB, probably due to the extended search, unique data structure, and output of multiple alignments.

### Comparison of the number of identified sites and runtime over various combinations of bulges and mismatches thresholds

We compared SWOffidner to CRISPRitz in a multi-threaded run over various combinations of bulges and mismatch thresholds. We excluded CALITAS from this comparison as it does not support a search with separate thresholds for bulges and mismatches, which is the common usage of CRISPR off-target search.^9^^,^^10^ In this comparison, we examined the number of identified off-target sites and the runtime for searches over thresholds combinations of *x* bulges and *y* mismatches where 1≤x≤2 and 1≤y≤5.

**Figure 4:**
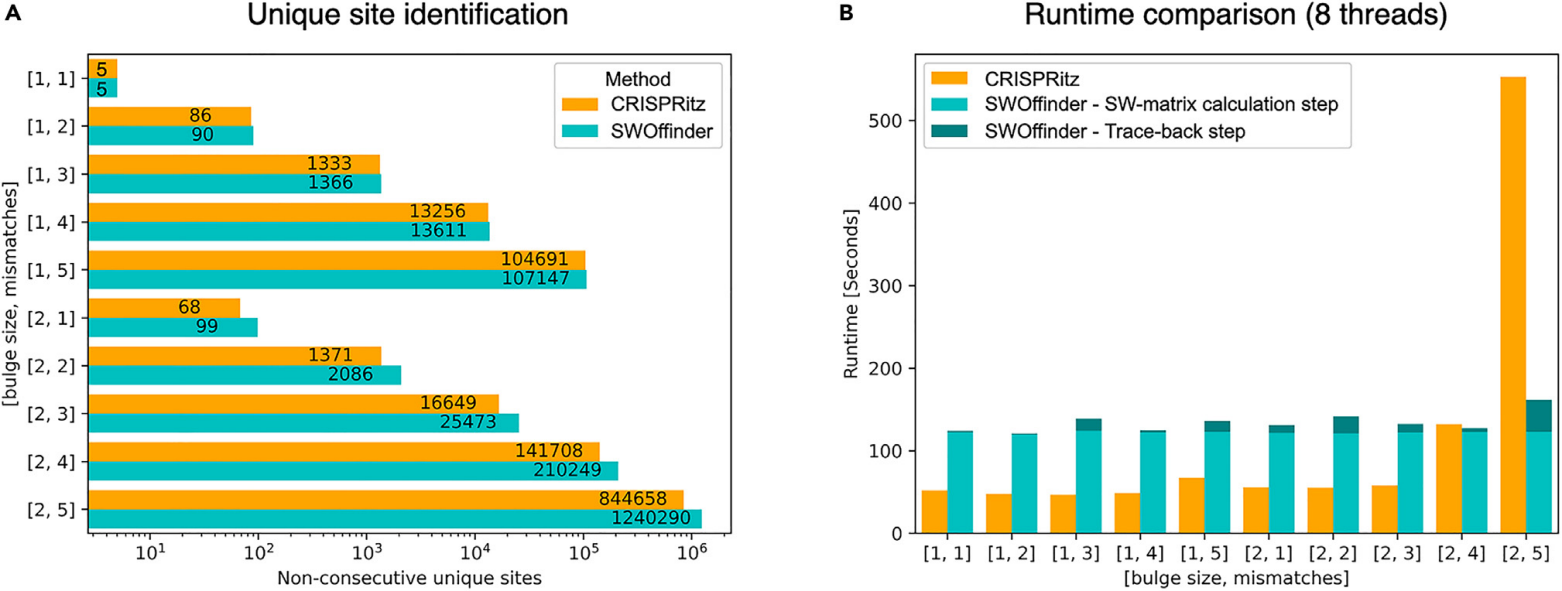
A comparison of the number of unique off-target sites found and the runtime between SWOffinder and CRISPRitz over various combinations of bulges and mismatches thresholds We did not compare to CALITAS in this benchmark as it does not support a search with a combination of separate thresholds. (A) A comparison of the unique off-target sites that were found. Note that the x-axis is on a logarithmic scale. (B) A comparison of the runtime. Note that the runtime of SWOffinder trace-back step was calculated by reducing the runtime of the SWOffinder SW-matrix calculation step from the total runtime.

The results show that for all comparisons SWOffider finds a greater number of unique off-target sites compared to CRISPRitz (Figure 4A). For example, for the combination of up to 1 bulge and 2 mismatches, SWOffinder found 90 sites while CRISPRitz found only 86. In the greater thresholds, the nominal difference in the number of unique sites is greater. For example, for the combination of up to 2 bulges and 5 mismatches, SWOffider found 1,240,290 non-consecutive unique sites compared to only 844,658 found by CRISPRitz.

In terms of runtime, we see that both CRISPRitz and SWOffinder have comparable runtime performances of 1−2 minutes over the human genome for small mismatch and bulge thresholds (Figure 4B). For combinations of up to 1 bulge and combinations of up to 2 bulges with up to 3 mismatches, CRISPRitz is outperforming SWOffinder, which achieves an almost constant runtime of 2 minutes due to the operation of step 1—the SW dynamic programming (DP) matrix filling that is the same for all thresholds. Strikingly, when we tested the combinations of up to 2 bulges and 4 or 5 mismatches, the trend changed. For example, for the combination of up to 2 bulges and 5 mismatches, SWOffinder was much faster than CRISPRitz with a runtime of 161.6 seconds compared to 552.62. This change in trends shows the advantage of using the two-step procedure in SWOffidner, where the SW-matrix calculation step is constant for all thresholds, and the trace-back step is negligible for most parameter combinations, including the more challenging ones. Another advantage of SWOffinder compared to CRISPRitz lies in its memory utilization. While SWOffinder is robust to the number of threads, CRISPRitz’s memory utilization increases with the number of threads. For example, for the combination of up to 2 bulges and 5 mismatches, the maximum memory usage of SWOffinder was slightly more than 2GB for both 1- and 8-threaded instances, while the usage of CRISPRitz was 3.3GB and 16.7GB, respectively. As a result, CRISPRitz’s memory utilization may become prohibitive with more threads.

## Discussion

In this work, we developed SWOffinder to enable a fast and accurate search of off-target sites defined by an edit-distance threshold, and separate thresholds for mismatches, and bulges. We modified the SW algorithm for local alignment and developed a novel post-processing step to find off-target sites with separate user-specified operation-specific thresholds. Our results show that SWOffinder finds more sites than existing off-target search methods. In addition, in our benchmarks, we show that even without genome indexing or unique data structures, SWOffinder achieves state-of-the-art performance in terms of runtime and memory utilization, as it runs across the human genome in only a few minutes with low memory usage.

The novelty in our approach lies in our novel modification of SW alignment and our novel post-processing approach. Our new post-processing algorithm has efficient runtime due to its DP implementation, and the fact that it runs on a limited set of off-target sites—only those that passed the edit-distance threshold as found by SW alignment. The new features we developed for searching under versatile criteria are much more relevant than the search enabled by current off-target search methods, as prior biological knowledge demonstrated that at most two bulges, or even just one bulge, can occur in an off-target site as opposed to tolerance to multiple mismatches.^11^

Our study may be extended in the future in several promising directions. First, SWOffinder can be extended to consider genetic variants to enable off-target search across individual genomes. This feature can be easily incorporated in both SW alignment and our novel post-processing approach, as it only affects the match and mismatch operations, which can be supported by encoding multiple nucleotides instead of just one. Second, we hope to speed up SWOffinder by utilizing dedicated hardware. Previous studies have shown that SW alignment can run much faster by implementing and running it on GPUs or even FPGAs,^12^ so similar improvements are expected for SWOffinder. Third, a possible solution to obtain all possible alignments in one pass of filling the DP matrix is to extend the recursive formula to include the number of bulges, and the number of mismatches. In this way, the matrix encodes whether an alignment of the number of bulges and mismatches exists. This comes at the cost of increasing the asymptotic runtime complexity by a factor of the number of allowed bulges and the number of allowed mismatches. Fourth, we plan to extend SWOffinder to run efficiently on multiple sgRNAs by reading the genome and traversing it only once for all sgRNAs.

To conclude, by addressing one of the major concerns associated with CRISPR/Cas9 technology, the possibility of unintended genetic modifications, SWOffinder promises to enhance the safety and accuracy of gene-editing processes. SWOffinder enables researchers to comprehensively identify potential off-target sites across the genome, allowing for thorough assessment and mitigation of off-target risks. Ultimately, the introduction of SWOffinder unlocks new avenues for precise genetic manipulation and paves the way for future advancements in the fields of medicine, agriculture, and biotechnology.

### Limitations of the study

The main limitation of SWOffinder is its worst-case asymptotic runtime complexity. The SW-matrix calculation step runs in O(mn), where the length of the sgRNA sequence is *m*, and the length of the genome is *n*. This runtime is independent of the user-specified thresholds. On the other hand, the trace-back step runs in time that depends linearly on the number of sites found in the SW-matrix calculation step, and exponentially on the number of bulges allowed. While the dependence on parameter values, and especially exponential dependence, is a disadvantage, as well as the dependence on the genomic content, in practice due to the number of sites found in the SW-matrix calculation step and our novel approach, which stops searching past the maximum effective edit distance, the runtime of the trace-back step is negligible compared to the SW-matrix calculation step. So, while SWOffinder does not benefit from a guaranteed polynomial runtime in the worst case, in practice, it runs in only a few minutes over the human genome for various combinations of thresholds and performs on par or outperforms existing methods depending on the thresholds.

## STAR★Methods

### Key resources table

**Table:** 

REAGENT or RESOURCE	SOURCE	IDENTIFIER
**Deposited data**		

Human reference genome NCBI build 38, GRCh38	The Genome Sequencing Consortium	https://hgdownload.soe.ucsc.edu/goldenPath/hg38/bigZips/

**Software and algorithms**		

CRISRitz	Cancellieri et al.^5^	https://github.com/pinellolab/CRISPRitz
CALITAS	Fennell et al.^6^	https://github.com/editasmedicine/calitas
SWOffinder	This paper	https://doi.org/10.5281/zenodo.10115908

### Resource availability

#### Lead contact

Further information and requests for resources should be directed to and will be fulfilled by the Lead Contact, Prof. Yaron Orenstein (yaron.orenstein@biu.ac.il).

#### Materials availability

This study did not generate new unique reagents.

#### Data and code availability

• The publicly available data that we used in this study is listed in the key resources table.
• All original code has been deposited at Zenodo and is publicly available as of the date of publication. DOI is listed in the key resources table.
• Any additional information required to reanalyze the data reported in this paper is available from the lead contact upon request.

### Method details

#### An overview of SWOffinder

SWOffinder utilizes the SW algorithm for local alignment, which is based on dynamic programming (DP).^13^ SWOffinder comprises of two steps:

(1) Using a novel version of SW alignment (Figure 2), we find all positions of sites under a user-specified edit-distance threshold.
(2) We apply a novel recursive procedure, which, given an end position of a potential off-target site obtained in the first step, finds the start position and the alignment of a site that satisfies user-specified separate thresholds with minimum edit distance (Algorithm 1).

#### SW-matrix calculation step details

Given a sgRNA sequence *a* of length *m*, and a DNA window *b* of length *n*, the first step of SWOffinder finds all end positions of sites in the DNA window that satisfy the edit-distance threshold, which we denote as maxE. There are some key differences in how we fill and use the DP matrix *M* of size (m+1)×(n+1) (Figure 2) compared to the original SW alignment.

• While SW alignment zeros the first row and column cells, we zero only the first row (M[0,j]=0 for 0≤j≤n). We do not zero the first column since it is impossible to have DNA bulges at the beginning of a sgRNA and DNA site alignment by definition.
• Since it is impossible to achieve separate thresholds for mismatches, insertions, and deletions with SW alignment, we use it for filtering sites that do not meet the edit-distance threshold. Therefore, we set unit penalty costs for all edit operations in the penalty matrix function (Equation 1) resulting in an asymptotic runtime complexity of O(mn).

(Equation 1) $$\begin{array}{l} M\left[i,j\right]=\min\left\{ \begin{array}{l} M\left[i-1,j-1\right]+s\left(i,j\right)\\ M\left[i,j-1\right]+1\\ M\left[i-1,j\right]+1 \end{array}\right.\\ s\left(i,j\right)=\left\{ \begin{array}{c} 1,a_{i}\neq b_{j}\\ 0,a_{i}=b_{j} \end{array}\right.\\ M\left[0,j\right]=0,0\leq j\leq n\\ M\left[i,0\right]=i,0<i\leq m \end{array}$$

• The main difference from SW alignment is how we use the DP matrix. While in general, the last column of the matrix is used to find an optimal local alignment end position, we use the last column to find end positions of all local alignments that have a total penalty cost smaller or equal to the edit-distance threshold, enabling us to find all off-target sites in a single fill of the DP matrix. Other methods, such as CALITAS,^6^ cannot find all off-target sites as they partition the genome into windows and find a global alignment in each window, which might miss multiple alignments in the same window.

#### Trace-back step details

In the second step of SWOffinder, given user-specified operation-specific thresholds, e.g. separate limits on the number of bulges and mismatches, SWOffinder returns an alignment that satisfies the separate thresholds with minimum edit distance, if there exists one, for any end position obtained in the first step. In SWOffinder, in addition to maxE, the edit-distance threshold which is used in the SW-matrix calculation step, the user may provide 3 additional thresholds on the number of mismatches and bulges:

(1) maxB: The number of bulges allowed in the alignment.
(2) maxM: The number of mismatches allowed in the alignment when no bulges exist in the alignment.
(3) maxMB: The number of mismatches allowed in the alignment when bulges exist in the alignment.

These versatile criteria enable a user to set diverse thresholds for mismatches and bulges, including distinct mismatch thresholds based on bulge presence or absence, which is common when processing and analyzing experimental off-target sites data.^14^

In addition to using the SW DP matrix in the SW-matrix calculation step to filter non-potential sites based on maxE, SWOffinder utilizes the matrix to terminate the alignment search when the matrix values indicate that it is impossible to achieve such alignment. To do so, we first calculate an effective max edit, effMaxE, that considers the separate operation-specific thresholds. effMaxE is calculated in each instance of the function POST to be either $\min\left(maxE,maxB+maxMB\right)$ when there are already bulges in the alignment or maxE when there are no bulges. Then, as described in lines 11−13 of Algorithm 1, the alignment search is terminated when the SW DP matrix value is greater than the allowed number of edits.

The runtime of the trace-back step is negligible compared to the SW-matrix calculation step in the most common cases for two main reasons: (i) it is applied to a small subset of the potential off-target sites that were obtained in the SW-matrix calculation step; and (ii) although the complexity of the trace-back step increases exponentially with the number of allowed bulges, this number is usually only 1 or 2 as prior CRISPR off-target studies showed that observing more than one DNA or RNA bulge is unlikely, even at the cost of multiple mismatches.^15^

#### Technical implementation details and features

##### Parallelism

Since the problem of searching off-target sites across a genome is easily parallelizable, to speed up the runtime of SWOffinder, we partition the given genome into multiple overlapping windows and then apply SWOffinder to each genome window in parallel. By the overlap, we ensure that SWOffinder does not miss any site in the border between the windows.

##### PAM search

SWOffinder supports off-target search with any PAM sequence and gives the user the flexibility to allow edits in the PAM sequence. If the user decides to prohibit mismatches or bulges in the PAM sequence, this can be easily achieved by modifying the SW recursive function in the relevant positions or by modifying the trace-back step of SWOffinder when the sgRNA pointer index (*i* in Algorithm 1) is in the PAM region. In the current implementation of SWOffinder, we implemented the second option. For clarity, we did not include this part in the pseudo-code of Algorithm 1.

##### Double-stranded search

As in other off-target search methods, SWOffinder supports off-target search on both the forward and reverse strands. To reduce I/O operations and avoid computing the reverse complement of the given genome when searching over the reverse strand, SWOffinder reads the genome only once and computes the reverse-complement of the sgRNA sequence instead of the genome. As a result, SWOffinder reverses the orientation of the PAM, which is taken into account when aligning the PAM sequence.

##### Versatile search options

There are multiple options for user-defined thresholds when searching for off-target sites by SWOffinder. Any combination of thresholds is allowed. In the most simple form, the user can search for all off-target sites up to a given edit-distance threshold. If additional thresholds are provided, i.e. for mismatches, mismatches with bulges, and/or bulges, the sites found under the edit-distance threshold are further filtered to meet the user-specified separate thresholds.

#### Finding an optimal site within a user-defined genome window

Most of the current state-of-the-art experimental techniques to detect off-target sites in the genome cannot identify the exact position of an off-target site within a sequencing read.^14^^,^^16^ Therefore, given a genome window (i.e., sequencing read) in which the edit occurred, a post-processing step of the experimental data aims to find an alignment to the sgRNA achieving minimum edit distance. In analogue, SWOffinder provides an option to find sites achieving the minimum edit distance within a genome window of a user-specified length. To enable this option, SWOffinder keeps two consecutive potential sites that were found in the trace-back step. If two sites are found in the same window, the site with the minimum edit distance is selected. In case of a tie, SWOffinder returns the first site.

#### Evaluation and comparison to existing off-target search methods

To evaluate the performance of SWOffinder and compare it to the state-of-the-art off-target search methods, CALITAS^6^ and CRISPRitz,^5^ we performed two types of evaluations: off-target site identification and runtime. We did not include Cas-OFFinder^3^ in the benchmarks and comparisons as its developers stated that their unpublished bulge-supported version is unstable.^17^ Indeed, previous studies included Cas-OFFinder in their benchmarks and comparisons and showed its inferior performance.^5^^,^^6^

For both CALITAS and CRISPRitz, we had to create a reference genome index prior to running the methods. In CRISPRitz, this is required for each combination of a PAM sequence and bulge size. We excluded the creation of the index from the runtime measurement.

We ran CALITAS with the following command line: calitas SearchReference -i <sgRNA with PAM> -r <genome reference and index path> -max-total-diffs <integer> -max-pam-mismatches <integer> -max-gaps-between-guide-and-pam <integer> -threads <integer>.

We ran CRISPRitz with the following command line: crispritz.py search <genome index path> <PAM file path> <sgRNAs list file path> <output path> -index -mm <integer> -bDNA <integer> -bRNA <integer> -r -th <integer>.

#### Benchmarking off-target sites identification

Although SWOffider is guaranteed to find all potential off-targets in a given genome thanks to the correctness of the SW algorithm, it is still necessary to validate the implementation of SWOffinder compared to other methods. To compare the methods in terms of off-target sites identification, we defined three tests:

(1) The number of unique sites, where a site is defined as the (chromosome, strand, end position) triplet.
(2) The number of non-consecutive unique sites, where sites with consecutive end positions are considered in the count as one site. This allows for avoiding scenarios where different sites are counted as unique only due to the permitted bulge size.
(3) The number of sites identified in a window of a site found by SWOffinder. We examined for each off-target site identified by SWOffinder whether other sites were found by existing methods in the genome window of the SWOffinder off-target site. The genome window is defined as the SWOffinder off-target site plus additional flanks of the size of the maximum bulge size allowed.

#### Runtime comparison

To compare the runtime performance of SWOffinder to state-of-the-art methods for CRISPR off-target search, we tested the runtime of the methods over various edit-distance thresholds, and combinations of mismatch and bulge thresholds, in both single- and multi-threaded runs. To conduct a fair comparison, we used the same AWS machine with 1^st^ generation Intel Xeon Platinum 8000 eight 3.1GHz cores, and 32GB RAM for all runtime tests, and reported the Elapsed (wall clock) time and Maximum Resident Set Size as measured by the Unix time -v command for runtime and maximum memory usage, respectively.
